# Appraisal of the overburden mass and boundary conditions on the rocking behaviour of the vertical spanning strip wall

**DOI:** 10.1007/s11012-024-01910-2

**Published:** 2024-12-19

**Authors:** Georgios Vlachakis, Carla Colombo, Dario Vecchio, Anastasios I. Giouvanidis, Paulo B. Lourenço

**Affiliations:** 1 ISISE, ARISE, Department of Civil Engineering, University of Minho, Guimarães, Portugal; 2 Department of Civil and Environmental Engineering, The University of Auckland, Auckland, New Zealand

**Keywords:** Out-of-plane collapse mechanism, Vertically spanning strip wall, Unreinforced masonry structures, Coefficient of restitution, Rocking dynamics, Boundary conditions

## Abstract

Unreinforced masonry structures are particularly vulnerable to seismic events. Specifically, local out-of-plane mechanisms form recurrently, posing a serious threat of collapse. Among them, a Vertical Spanning Strip Wall (VSSW) mechanism occurs when a portion of a wall is solely constrained at its top and base, experiencing out-of-plane bending across its height. During the formation of the mechanism and up to collapse, the VSSW presents a highly nonlinear dynamic behaviour governed by the presence of an overlying wall or diaphragm and a diversity of diaphragm-to-wall connections. The present work simulates the dynamics of a VSSW, accounting for the influence of the overburden mass and a variety of boundary conditions through a two-rigid-body single-degree-of-freedom model. In addition, this study examines the energy losses of the VSSW and provides closed-form expressions to estimate the angular coefficient of restitution either analytically for predictive purposes or experimentally for characterisation campaigns. Finally, a series of illustrative comparative free- and forced-rocking analyses highlight the importance of the overburden mass and boundary conditions on the dynamic stability of the VSSW.

## Introduction

Unreinforced masonry structures constitute a significant portion of our built environment, especially when considering heritage constructions [1]. Despite the societal demand for their safeguard, unreinforced masonry structures have shown to be particularly vulnerable to seismic actions [2–4]. Their vulnerability stems mainly from their high mass, weak connections among structural elements and the brittle behaviour of masonry [5]. As a result, cracks develop rather easily during earthquakes, forming Out-Of-Plane (OOP) and In-Plane (IP) collapse mechanisms [6]. The former usually occurs when the connections among the structural elements are insufficient, whilst the latter requires a so-called “box-like” or integral structural behaviour. Most importantly, even though OOP mechanisms are more common and destructive than IP ones, their complex dynamic response is less understood [7, 8]. Among the OOP mechanisms, the simple overturning of a rocking wall is undoubtedly the weakest and therefore has attracted the highest attention in the literature [9–11]. Nonetheless, the second most vulnerable OOP mechanism is the Vertical Spanning Strip Wall (VSSW). Such a mechanism appears when a wall is restrained at its top and base. In fact, the Boundary Conditions (BCs) on top, together with the formation of three hinges along the wall’s height and their associated impact interfaces, make the VSSW mechanism a challenging topic of structural dynamics [12–16].

There is a scarcity of experimental studies dedicated to the VSSW [17–23], while several numerical and analytical models have been proposed to replicate its dynamic behaviour. More specifically, some formulations resort to rocking dynamics [10, 12, 13, 24, 25], while others adopt a series of assumptions on the dynamics of the system to simplify the problem and propose practical methodologies [26–29], such as the linearisation of the equation of motion and the kinematics [26, 28] or the adoption of multi-linear moment-rotation diagrams [26–29]. Recent works have extended the investigation to flexible interfaces [24] or flexible BCs [12, 27].

The quantification of the energy losses of the VSSW mechanism is another open issue, which has been addressed by analytical mechanics-based considerations or by direct experimental observations. Sorrentino et al. [13], Mehrotra and DeJong [24] and Prajapati et al. [12] estimated the angular Coefficient of Restitution (CoR) by resorting to impulsive dynamics and the conservation of momentum. More recent numerical studies either adopted the previous or utilised equivalent viscous damping models [10, 16, 26–30]. Nonetheless, comparisons of the dynamic response of numerical predictions with experiments have been successful only after careful calibration of the damping models [16, 27–29]. At the same time, a few campaigns have quantified the experimental energy losses of the VSSW [17, 20]; however, comparisons with the analytical predictions showed a mismatch [20].

Overall, previous studies have highlighted the influence of several factors on the response of the VSSW mechanism, namely the height of the intermediate hinge, the significance of the overburden load, the influence of the energy losses at impacts, and the effect of flexible BCs and interfaces.

The present work complements these studies and investigates in depth the influence of the BCs and the overburden mass on the seismic response of the VSSW. To this end, a refined model is developed to incorporate a parametrised position of the hinge on top of the VSSW and the presence of an overburden mass. The former generalisation allows the replication of a variety of symmetric and asymmetric BCs found in real-world VSSW structures [31], whereas currently available models restrain the BCs at predefined positions [10, 12, 13, 24–29]. Furthermore, the incorporation of an overburden mass essentially replicates an overlaying structure, the weight of the diaphragm, among others. Previous models usually employed an overburden force instead of a mass to simulate this aspect [10, 13, 24, 26, 28, 29]. However, a mass has greater implications on the dynamics of the VSSW, as it contributes to both the inertia and momentum. Importantly, this work demonstrates through extensive comparative analyses how the aforementioned features influence the Equation of Motion (EoM), the hinge height, the instability angle, the energy losses, and the overall dynamic response of the VSSW.

The paper is organised as follows: Sect. 2 presents the formulation of the model, the description of the system’s kinematics and the derivation of the EoM. Section 3 discusses the energy losses of the system and proposes: (i) an analytical formula to estimate the angular CoR based on impulsive dynamics, and (ii) a formula to extract the angular CoR from experimental response-histories. Section 4 illustrates the influence of the BCs and overburden mass on the dynamic response of the VSSW, through free- and forced-rocking analyses. Finally, Sect. 5 summarises the conclusions of the study.

## Model formulation

### Geometry and kinematics

**Fig. 1:**
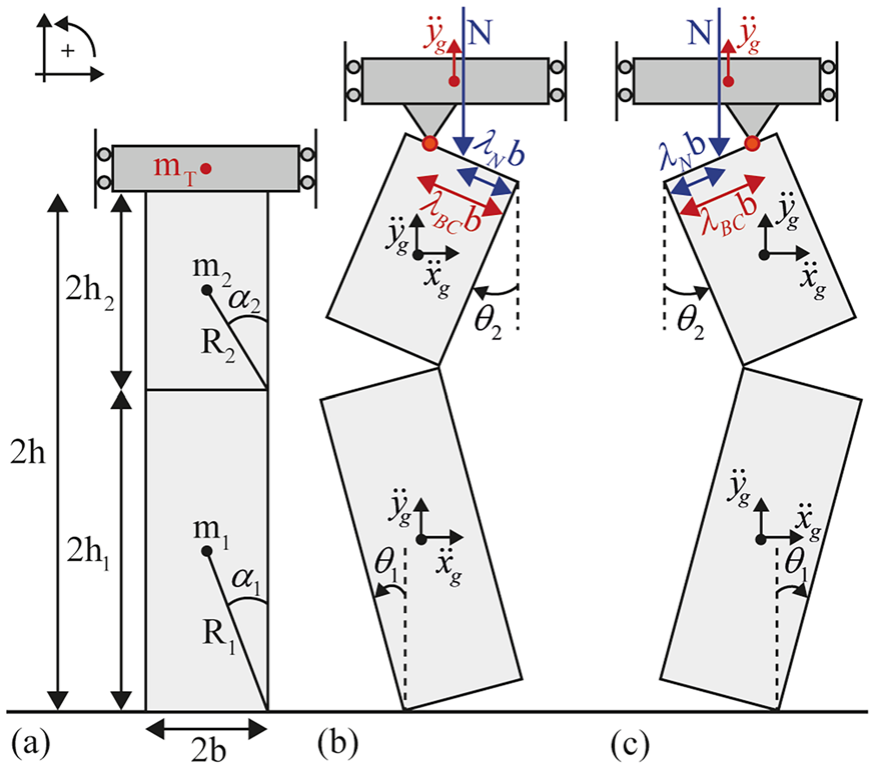
Scheme of the vertical spanning strip wall: **a** geometry and displaced configuration with boundary conditions for **b** positive (counter-clockwise) and **c** negative (clockwise) rotation of the lower body “1”

Consider the VSSW depicted in Fig. 1**a** with width 2*b*, height 2*h*. Upon ground excitation, assuming no sliding at the contact interfaces and given the horizontal restraint on top of the wall, three hinges are formed for the activation of the VSSW mechanism: (i) at the base of the wall, (ii) at an intermediate height $2h_{1}$ (relative to the base), and (iii) at the top of the wall. As a result, the wall is divided into two distinct rigid bodies (Fig. 1**a**): the lower body “1” and the upper body “2”, with heights $2h_{1}$ and $2h_{2}$, slenderness angles $\alpha_{1}$ and $\alpha_{2}$, and diagonal distances $R_{1}$ and $R_{2}$, respectively. The aforementioned geometry can be fully described using a set of three parameters, e.g. the half width $b$ and the two half-heights $h_{1}$ and $h_{2}$, or the two slenderness angles $\alpha_{1}$ and $\alpha_{2}$, and the diagonal distance $R_{1}$. Similar to Sorrentino et al. [13], this study adopts the latter set of variables.

**Fig. 2:**
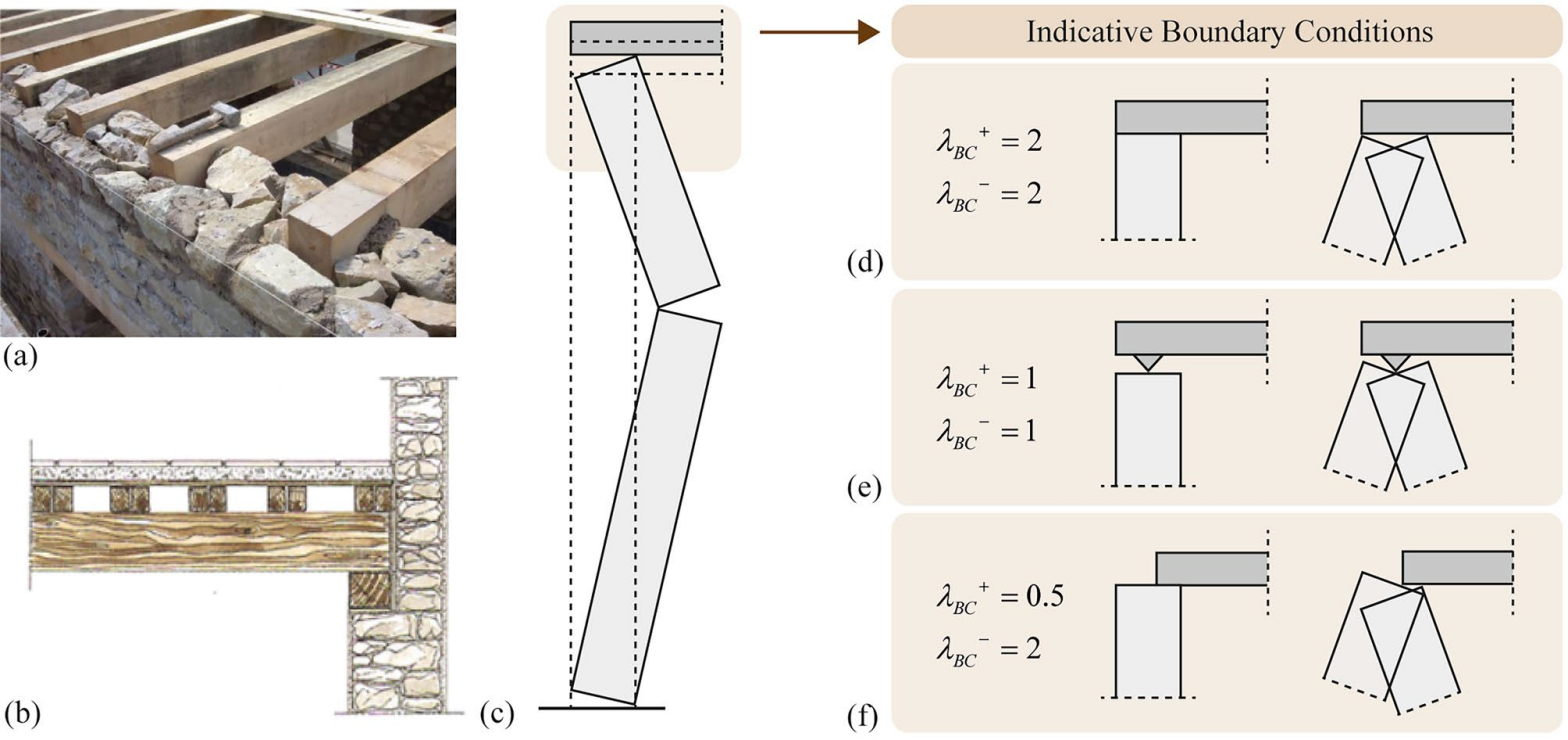
Boundary conditions on top of the vertical spanning strip wall: **a** structural detail during the construction stage (reproduced from [33] with permission from Springer Nature), **b** schematic view of the diaphragm-wall connection (reproduced from [31] with permission from Elsevier), **c** schematic view of the displaced wall, and **d**–**f** view of illustrative boundary conditions that the present model replicates

The position of the hinge at the top of the wall is usually assumed to be either at the upper left (Fig. 1**b**) or right corner of the body “2” (Fig. 1**c**) depending on the sign of rotation [10, 13, 28], or at the centre of the wall [29]. Nevertheless, the position of the hinge is directly related to the Boundary Conditions (BCs) and the connection of the wall with the horizontal diaphragm/constraint. As a matter of fact, a wide diversity of BCs can be found in real structures [31, 32], with Fig. 2**a**–**b** showing two cases where the diaphragm rests on a limited portion of the wall. Considering this diversity, the current model parametrises the position $\lambda_{BC} \in \left[ {0,2} \right]$ of the top hinge (Fig. 1**b**–**c**). This allows replicating a variety of BCs using different values of $\lambda_{BC}$, with Fig. 2**b**–**f** illustrating three indicative examples: (i) “clamped” with $\lambda_{BC}^{ + } = \lambda_{BC}^{ - } = 2$, where pivoting occurs at the upper corners (Fig. 2**d**), (ii) “pinned” with $\lambda_{BC}^{ + } = \lambda_{BC}^{ - } = 1$, where pivoting occurs at the centre (Fig. 2**e**), and (iii) asymmetric with $\lambda_{BC}^{ + } = 0.5$ and $\lambda_{BC}^{ - } = 2$, where pivoting occurs at the upper corner of the VSSW for counter-clockwise rotation and at the corner of the diaphragm for clockwise rotation of the upper body “2”, respectively (Fig. 2**f**). The superscripts indicate the sign of rotation of the lower body “1” of the VSSW, i.e. “ + ” for positive counter-clockwise rotation and “–” for negative clockwise rotation.

The present model considers also the influence of an overburden mass ($m_{T}$) and an overload (${\rm N}$) acting at the top of the wall (Fig. 1**b**–**c**). These replicate the weight of any overlaying structure resting on top of the wall, such as an upper storey, an overlaying parapet or a diaphragm. Previous models in the literature have focused solely on the overload [10, 13]. Nevertheless, in practice both scenarios may occur. Their influence on the VSSW appears comparable, although the mass has additional inertia and momentum directly affecting the system’s dynamics. Note that the overburden mass $m_{T}$ is assumed to act at the hinge point at the top and it is controlled by the parameter $\lambda_{BC}$. Similarly, the overburden load ${\rm N}$ is parametrised by the distance $\lambda_{N} \in \left[ {0,2} \right]$ shown in Fig. 1**b**–**c**.

Assuming that the two bodies are rigid, can uplift and rock but not slide or rebound, the pure rocking motion of the VSSW can be captured by a single degree of freedom. This is assumed to be the angular rotation $\theta_{1}$ of the lower body “1”, with the angular rotation of the upper body “2” $\theta_{2}$ being related to the former through the constraint condition introduced by the intermediate and top hinges:

1 $$\theta_{2} = \arcsin C_{1} - \varphi$$

with:

2 $$\varphi = {\text{sgn}} \theta_{1} \arctan \left( {\frac{{\lambda_{BC} }}{2}\tan \alpha_{2} } \right)$$

The term $C_{1}$ is given in the Appendix 1. Similarly, the angular velocities $\dot{\theta }_{1}$ and $\dot{\theta }_{2}$ of the two bodies can also be related as:

3 $$\dot{\theta }_{2} = \frac{{dC_{1} }}{{d\theta_{1} }}\frac{1}{{\sqrt {1 - C_{1}^{2} } }}\dot{\theta }_{1}$$

where $\frac{{dC_{1} }}{{d\theta_{1} }}$ is provided in the Appendix 1.

### Equation of motion (EoM)

The EoM of the system of Fig. 1 is derived using the Lagrange’s equation:

4 $$\frac{d}{dt}\left( {\frac{{\partial \left( {Z - V} \right)}}{{\partial \dot{\theta }_{1} }}} \right) - \frac{{\partial \left( {Z - V} \right)}}{{\partial \theta_{1} }} = \Gamma_{\rm N} + \Gamma_{g}$$

where $V$ and $Z$ represent the potential and kinetic energies of the system, while $\Gamma_{\rm N}$ and $\Gamma_{g}$ are the work done by the overload force ${\rm N}$ and the inertia forces due to the horizontal and vertical components of the ground acceleration $\ddot{x}_{g}$ and $\ddot{y}_{g}$, respectively. The potential energy $V$ is:

5 $$V = gR_{1} \left[ {\left( {m_{1} + 2m_{2} + 2m_{T} } \right)\cos \left( {\alpha_{1} - \left| {\theta_{1} } \right|} \right) + m_{2} \frac{{\sin \alpha_{1} }}{{\sin \alpha_{2} }}\cos \left( {\alpha_{2} - \left| {\theta_{2} } \right|} \right) + 2m_{T} \frac{{\sin \alpha_{1} }}{{\tan \alpha_{2} }}\frac{{\cos \theta_{2} }}{\cos \varphi }} \right]$$

where $m_{1}$ and $m_{2}$ are the mass of the bodies “1” and “2”, respectively, and $g$ is the acceleration of gravity. The kinetic energy $Z$ reads:

6 $$Z = \frac{1}{2}R_{1}^{2} \dot{\theta }_{1}^{2} \left[ {m_{1} + m_{2} \left( {C_{2}^{2} + C_{3}^{2} } \right) + m_{T} C_{4}^{2} } \right] + \frac{1}{2}\dot{\theta }_{1}^{2} \left[ {I_{G1} + I_{G2} \left( {\frac{{dC_{1} }}{{d\theta_{1} }}} \right)^{2} \frac{1}{{1 - C_{1}^{2} }}} \right]$$

with $I_{G1}$ and $I_{G2}$ being the moment of inertia of the bodies “1” and “2” with respect to their centre of gravity, while the terms $C_{2}$, $C_{3}$ and $C_{4}$ are provided in the Appendix 1. The virtual work of the superimposed load $\Gamma_{\rm N}$ is:

7 $$\delta \Gamma_{\rm N} = - NR_{1} \left[ {2{\text{sgn}} \theta_{1} \sin \left( {\alpha_{1} - \left| {\theta_{1} } \right|} \right) - \frac{{\sin \alpha_{1} }}{{\sin \alpha_{2} }}\frac{{dC_{1} }}{{d\theta_{1} }}\frac{1}{{\sqrt {1 - C_{1}^{2} } }}\left( {{\text{sgn}} \theta_{1} \lambda_{N} \sin \alpha_{2} \cos \theta_{2} + 2\cos \alpha_{2} \sin \theta_{2} } \right)} \right]\delta \theta_{1}$$

and the virtual work of the inertia forces $\Gamma_{g}$ reads:

8 $$\delta \Gamma_{g} = \left\{ { - \ddot{x}_{g} R_{1} \left[ {m_{1} \cos \left( {\alpha_{1} - \left| {\theta_{1} } \right|} \right) - m_{2} C_{2} } \right] + \ddot{y}_{g} R_{1} \left[ {m_{1} {\text{sgn}} \theta_{1} \sin \left( {\alpha_{1} - \left| {\theta_{1} } \right|} \right) + m_{2} C_{3} + m_{T} C_{4} } \right]} \right\}\delta \theta_{1}$$

After expanding Eq. (4), the following non-linear second-order differential equation is obtained:

9 $$C_{A} \ddot{\theta }_{1} + C_{S} \dot{\theta }_{1}^{2} = - R_{1} C_{H} \ddot{x}_{g} + R_{1} C_{V} \left( {\ddot{y}_{g} - g} \right) - R_{1} C_{N} N$$

where the terms $C_{A}$, $C_{S}$, $C_{H}$, $C_{V}$ and $C_{N}$ are:

10a $$C_{A} = R_{1}^{2} \left[ {m_{1} + m_{2} \left( {C_{2}^{2} + C_{3}^{2} } \right) + m_{T} C_{4}^{2} } \right] + I_{G1} + I_{G2} \left( {\frac{{dC_{1} }}{{d\theta_{1} }}} \right)^{2} \frac{1}{{1 - C_{1}^{2} }}$$

10b $$C_{S} = R_{1}^{2} \left[ {m_{2} \left( {C_{2} \frac{{dC_{2} }}{{d\theta_{1} }} + C_{3} \frac{{dC_{3} }}{{d\theta_{1} }}} \right) + m_{T} C_{4} \frac{{dC_{4} }}{{d\theta_{1} }}} \right] + \frac{{I_{G2} }}{{\left( {1 - C_{1}^{2} } \right)^{2} }}\left[ {\frac{{d^{2} C_{1} }}{{d\theta_{1}^{2} }}\frac{{dC_{1} }}{{d\theta_{1} }}\left( {1 - C_{1}^{2} } \right) + \left( {\frac{{dC_{1} }}{{d\theta_{1} }}} \right)^{3} C_{1} } \right]$$

10c $$C_{H} = m_{1} \cos \left( {\alpha_{1} - \left| {\theta_{1} } \right|} \right) - m_{2} C_{2}$$

10d $$C_{V} = m_{1} {\text{sgn}} \theta_{1} \sin \left( {\alpha_{1} - \left| {\theta_{1} } \right|} \right) + m_{2} C_{3} + m_{T} C_{4}$$

10e $$C_{N} = 2{\text{sgn}} \theta_{1} \sin \left( {\alpha_{1} - \left| {\theta_{1} } \right|} \right) - \frac{{\sin \alpha_{1} }}{{\sin \alpha_{2} }}\frac{{dC_{1} }}{{d\theta_{1} }}\frac{1}{{\sqrt {1 - C_{1}^{2} } }}\left( {{\text{sgn}} \theta_{1} \lambda_{N} \sin \alpha_{2} \cos \theta_{2} + 2\cos \alpha_{2} \sin \theta_{2} } \right)$$

Note that the overburden mass $m_{T}$ is included both in the inertia term $C_{A}$ and the centrifugal and Coriolis term $C_{S}$.

### Rocking initiation, intermediate hinge height and instability angle

The minimum horizontal ground acceleration $\left| {\ddot{x}_{g} } \right|_{\min }$ capable of initiating rocking motion can be computed by setting $\theta_{1} = \dot{\theta }_{1} = \ddot{\theta }_{1} = 0$ in Eq. (9):

11 $$\left| {\ddot{x}_{g} } \right|_{\min } = \tan \alpha_{1} \frac{{\left( {g - \ddot{y}_{g} } \right)\left[ {m_{1} + m_{2} \left( {2 + \frac{{\tan \alpha_{2} }}{{\tan \alpha_{1} }}} \right) + m_{T} \left( {2 + \lambda_{BC} \frac{{\tan \alpha_{2} }}{{\tan \alpha_{1} }}} \right)} \right] + N\left( {2 + \lambda_{N} \frac{{\tan \alpha_{2} }}{{\tan \alpha_{1} }}} \right)}}{{m_{1} + m_{2} }}$$

Notice that the overburden weight $m_{T} g$ and the overload force ${\rm N}$ have exactly the same influence on $\left| {\ddot{x}_{g} } \right|_{\min }$ when $\lambda_{BC} = \lambda_{N}$ and $\ddot{y}_{g} = 0$.

**Fig. 3:**
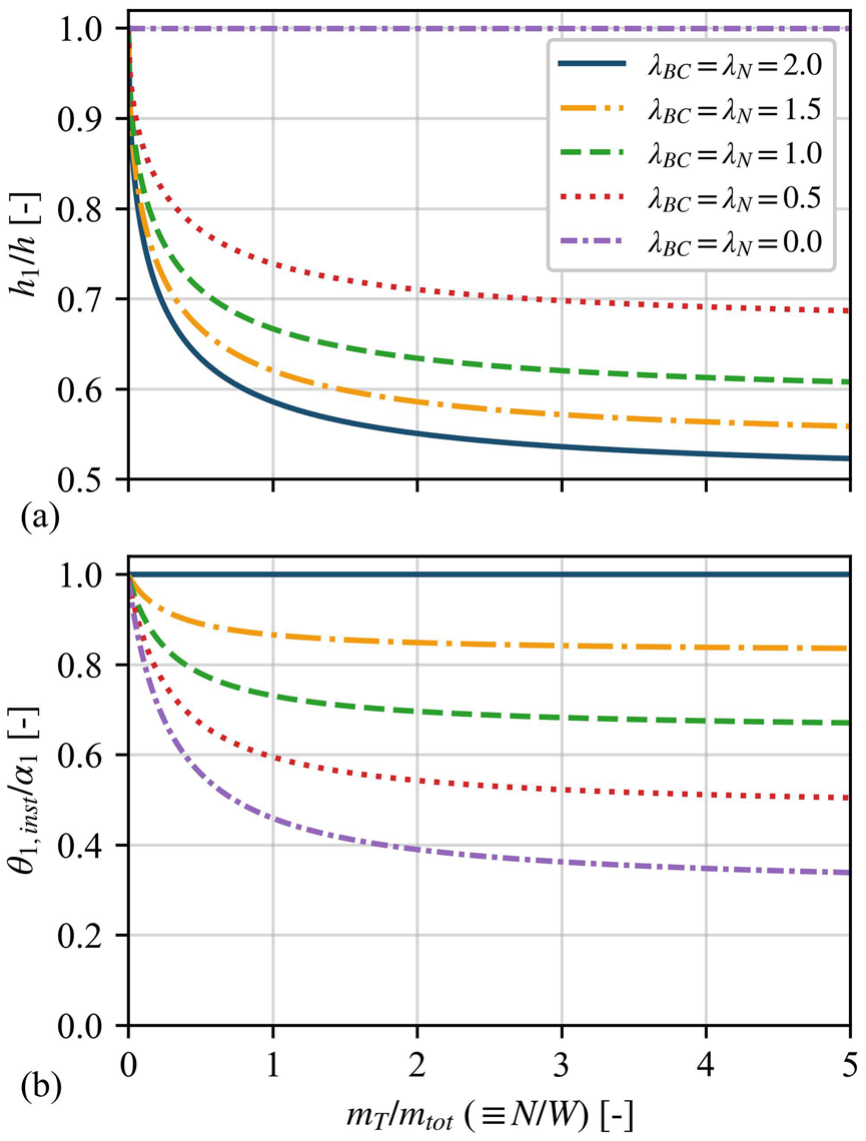
**a** Intermediate hinge height as a function of the overburden mass (or load) for various boundary (overload) conditions, and **b** instability angle as a function of the overburden mass (or load) for various boundary (overload) conditions, assuming a fixed hinge height ${h_{1} / h} = 0.7$

For walls without tensile strength (such as dry-joint masonry), the height of the intermediate hinge $2h_{1}$ can be computed as the height that minimises Eq. (11) [13]. Figure 3**a** plots the intermediate hinge height $2h_{1}$ normalised by the height of the wall $2h$ as a function of the overburden mass $m_{T}$ normalised by the total mass of the wall $m_{tot}$ (i.e. $m_{tot} = m_{1} + m_{2}$), which is equivalent to the overload ${\rm N}$ normalised by the weight of the wall $W$ (i.e. $W = m_{tot} g$). Furthermore, Fig. 3**a** depicts the influence of the overburden’s mass position $\lambda_{BC}$ (and equivalently the overload’s position $\lambda_{N}$, assuming $\lambda_{BC} = \lambda_{N}$) on the hinge height. Overall, Fig. 3**a** shows that the intermediate hinge height changes drastically for small ${{m_{T} } /{m_{tot} }}$ (or $N / W$) values while it asymptotically stabilises for higher ${{m_{T} } /{m_{tot} }}$ (or $N / W$) values. Moreover, the position of the overburden mass (or overload) has an inverse effect on the hinge height, with the lower values of $\lambda_{BC}$ (or $\lambda_{N}$) causing an increase in the hinge height. As a result, the intermediate hinge height drops to almost half of the wall’s height for very high overburden mass (or overload) and large values of $\lambda_{BC}$ (or $\lambda_{N}$). On the contrary, for very low overburden mass (or overload) and regardless of the value of $\lambda_{BC}$ (or $\lambda_{N}$), the intermediate hinge height asymptotically tends to one, which resembles the single rocking block configuration. Finally, it is worth noting that, based on experimental evidence, the intermediate hinge height ranges between 0.5 and 0.75 the height of the wall [18, 20–23]. Figure 3**a** indicates a comparable range of values, while discrepancies between the experimental evidence and the proposed model may arise due to the presence of joints at discrete heights, the presence of mortar with tensile strength, or any other sources of imperfection.

Furthermore, the static instability angle ($\theta_{1,inst}$) of a VSSW can be computed by setting $\dot{\theta }_{1} = \ddot{\theta }_{1} = \ddot{x}_{g} = \ddot{y}_{g} = 0$ in Eq. (9) and solving for $\theta_{1}$. In this context, Fig. 3**b** illustrates the instability angle $\theta_{1,inst}$ normalised by the slenderness angle of body “1” $\alpha_{1}$, as a function of the normalised overburden mass ${{m_{T} } /{m_{tot} }}$ (and equivalently the overload $N / W$), for various positions of the overburden mass $\lambda_{BC}$ (and equivalently positions of the overload $\lambda_{N}$, assuming $\lambda_{BC} = \lambda_{N}$) for a fixed intermediate hinge height ${h_{1} / h} = 0.7$. Firstly, Fig. 3**b** shows that $\theta_{1,inst}$ is equal to $\alpha_{1}$ only when $\lambda_{BC} = \lambda_{N} = 2$ or for zero overburden mass (or overload), whereas in any other case, $\theta_{1,inst}$ is less than $\alpha_{1}$. In general, this counterintuitive behaviour stems from the maximum stabilising lever arm of the overburden mass (or overload) when $\lambda_{BC} = 2$ (or $\lambda_{N} = 2$), relative to the intermediate hinge. This effect diminishes as $\lambda_{BC}$ (or $\lambda_{N}$) decreases. More specifically, $\theta_{1,inst}$ decreases rapidly for low ${{m_{T} } /{m_{tot} }}$ (or $N / W$) values, and it plateaus for high ${{m_{T} } /{m_{tot} }}$ (or $N / W$) values. Furthermore, the position of the overburden mass (overload) influences strongly $\theta_{1,inst}$, with the lower values of $\lambda_{BC}$ (or $\lambda_{N}$) resulting in smaller $\theta_{1,inst}$.

## Energy dissipation—Coefficient of restitution (CoR)

During rocking motion, when $\theta_{1}$ changes sign, simultaneous impacts occur at the interface between the two bodies and their BCs, resulting in sudden energy losses. This complex damping phenomenon is commonly accounted for using the angular CoR $e_{\theta }$, which relates the pre-impact with the post-impact angular velocities $e_{\theta } = {{\dot{\theta }_{1}^{ + } }}/ {\dot{\theta }_{1}^{ - }}$. It is understood that $e_{\theta }$ is merely a phenomenological representation that considers implicitly the complex impact phenomena taking place at the contact interfaces and the radiation damping occurring within the bodies. Nonetheless, the simplicity of the angular CoR makes it particularly convenient from a structural perspective [34]. Therefore, this Section presents two ways to compute the angular CoR: (i) using analytical impulsive dynamics, and (ii) from experimental measurements. The former is useful for predictive purposes, while the latter is beneficial for characterisation, model assessment, and calibration after experimental campaigns.

### Analytical angular coefficient of restitution

This Section provides an analytical expression of the angular CoR $e_{\theta }$ of the VSSW of Fig. 1. To derive it, this work adopts the common assumptions of impulsive rocking dynamics, which can be summarised as follows [13, 35–37]: (i) instantaneous duration of impact, (ii) change of angular velocity with no variation of rotation, (iii) sticking impact (i.e. rocking motion is sustained, without sliding, bouncing or free-flight), and (iv) non-impulsive forces (e.g. body weights, overload force, reaction forces etc.) are negligible to the outcome of impact. Within this framework, the angular CoR is computed assuming the conservation of angular momentum over the pivot point of the post-impact configuration.

**Fig. 4:**
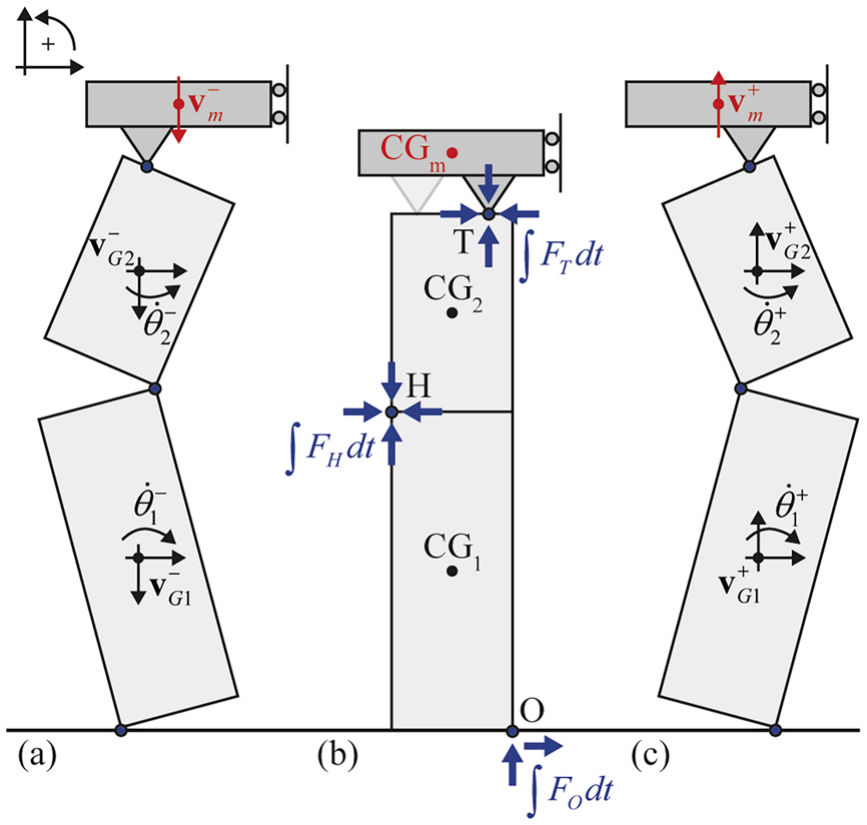
Impact instances scheme and associated impulses from **a** a counter-clockwise rotation to **b** impact and **c** clockwise rotation of the lower body

Figure 4 illustrates the examined impact scheme, depicting the VSSW: (i) before impact where bodies “1” and “2” have linear (${\mathbf{v}}_{G1}^{ - }$, ${\mathbf{v}}_{G2}^{ - }$) and angular ($\dot{\theta }_{1}^{ - }$, $\dot{\theta }_{2}^{ - }$) pre-impact velocities with the top mass having only linear (${\mathbf{v}}_{m}^{ - }$) pre-impact velocity (Fig. 4**a**), (ii) during impact where contact impulses ($\int {F_{O} dt}$, $\int {F_{H} dt}$, $\int {F_{T} dt}$) at the impact points “O”, “H” and “T” are introduced (Fig. 4**b**), and iii) after impact where bodies “1” and “2” have linear (${\mathbf{v}}_{G1}^{ + }$, ${\mathbf{v}}_{G2}^{ + }$) and angular ($\dot{\theta }_{1}^{ + }$, $\dot{\theta }_{2}^{ + }$) post-impact velocities with the top mass having only linear (${\mathbf{v}}_{m}^{ + }$) post-impact velocity (Fig. 4**c**). The conservation of angular momentum over point “O” reads:

12 $$\begin{gathered} H_{O}^{ - } = H_{O}^{ + } \Rightarrow \hfill \\ I_{G1} \dot{\theta }_{1}^{ - } + m_{1} \left( {{\mathbf{r}}_{G1,O} \times {\mathbf{v}}_{G1}^{ - } } \right) + I_{G2} \dot{\theta }_{2}^{ - } + m_{2} \left( {{\mathbf{r}}_{G2,O} \times {\mathbf{v}}_{G2}^{ - } } \right) + m_{T} \left( {{\mathbf{r}}_{m,O} \times {\mathbf{v}}_{m}^{ - } } \right) = \hfill \\ = I_{G1} \dot{\theta }_{1}^{ + } + m_{1} \left( {{\mathbf{r}}_{G1,O} \times {\mathbf{v}}_{G1}^{ + } } \right) + I_{G2} \dot{\theta }_{2}^{ + } + m_{2} \left( {{\mathbf{r}}_{G2,O} \times {\mathbf{v}}_{G2}^{ + } } \right) + m_{T} \left( {{\mathbf{r}}_{m,O} \times {\mathbf{v}}_{m}^{ + } } \right) \hfill \\ \end{gathered}$$

where ${\mathbf{r}}_{G1,O}$, ${\mathbf{r}}_{G2,O}$ and ${\mathbf{r}}_{m,O}$, are the relative position vectors of the Centre of Gravity (CG) of body “1”, the CG of body “2” and the CG of the top mass with respect to the impact point O, while the superscript sign symbols “–” and “ + ” refer to the time-instants before and after the impact, respectively. Here, two assumptions related to the mass on top are adopted: (i) the position of its CG_m_ at the time-instant of impact is assumed to be above the CG_1_, CG_2_ of the VSSW, and (ii) it has negligible rotational momentum. These assumptions are valid when the overburden mass stems from a wall resting on top of the VSSW (e.g. a parapet, an upper storey or a gable) but entail an approximation when the overburden mass is due to a diaphragm with distributed mass across its span. In the latter case, one may follow the same procedure and include the additional term of the rotational inertia of the top mass together with its angular velocity (related also to its boundary condition). Nevertheless, such analysis merits further investigation which is beyond the scope of the present work.

After expanding Eq. (12), the angular CoR $e_{\theta }$ becomes:13where, again, the superscript sign symbols “–” and “ + ” refer to the time-instant prior and after impact, respectively. In particular, if the height of the intermediate hinge remains the same before and after impact, the superscript sign concerns solely the term related to $\lambda_{BC}$ (i.e. $m_{T} R_{1}^{2} \left( {\cos^{2} \alpha_{1} \lambda_{BC} \frac{{\tan \alpha_{2} }}{{\tan \alpha_{1} }} + 2\sin^{2} \alpha_{1} } \right)$ in Eq. (13)). Moreover, in the case of symmetric BCs (e.g. Figure 2**d**, **e**) and the same intermediate hinge height before and after impact, the superscript sign is redundant. Contrary to the mass on top $m_{T}$, the overload ${\rm N}$ does not influence the angular CoR. Instead, the different BCs play a role only if there is a mass on top, since the term $\lambda_{BC}$ affects solely the term related to $m_{T}$. In addition, Eq. (13) confirms the equation of Sorrentino et al. [13] when $m_{T} = 0$.

Considering that the total energy of the system ($Z + V$) cannot increase and that the potential energy remains constant at impact implies that before and after impact

14 $$Z^{ + } \le Z^{ - } \Rightarrow e_{\theta }^{2} \le \frac{{\left\langle {C_{5} } \right\rangle^{ - } }}{{\left\langle {C_{5} } \right\rangle^{ + } }}$$

with the term $C_{5}$ given in the Appendix 1. This condition is always satisfied for symmetric configurations (e.g. Figure 2**d**,**e**), but not necessarily for asymmetric ones (e.g. Figure 2**f**). Nonetheless, for any VSSW system, one can compute the angular CoR $e_{\theta }$ using Eq. (13), check the energy condition using Eq. (14), and if the condition is not satisfied, reduce the angular CoR $e_{\theta }$ to:

15 $$e_{\theta } = \sqrt {\frac{{\left\langle {C_{5} } \right\rangle^{ - } }}{{\left\langle {C_{5} } \right\rangle^{ + } }}}$$

**Fig. 5:**
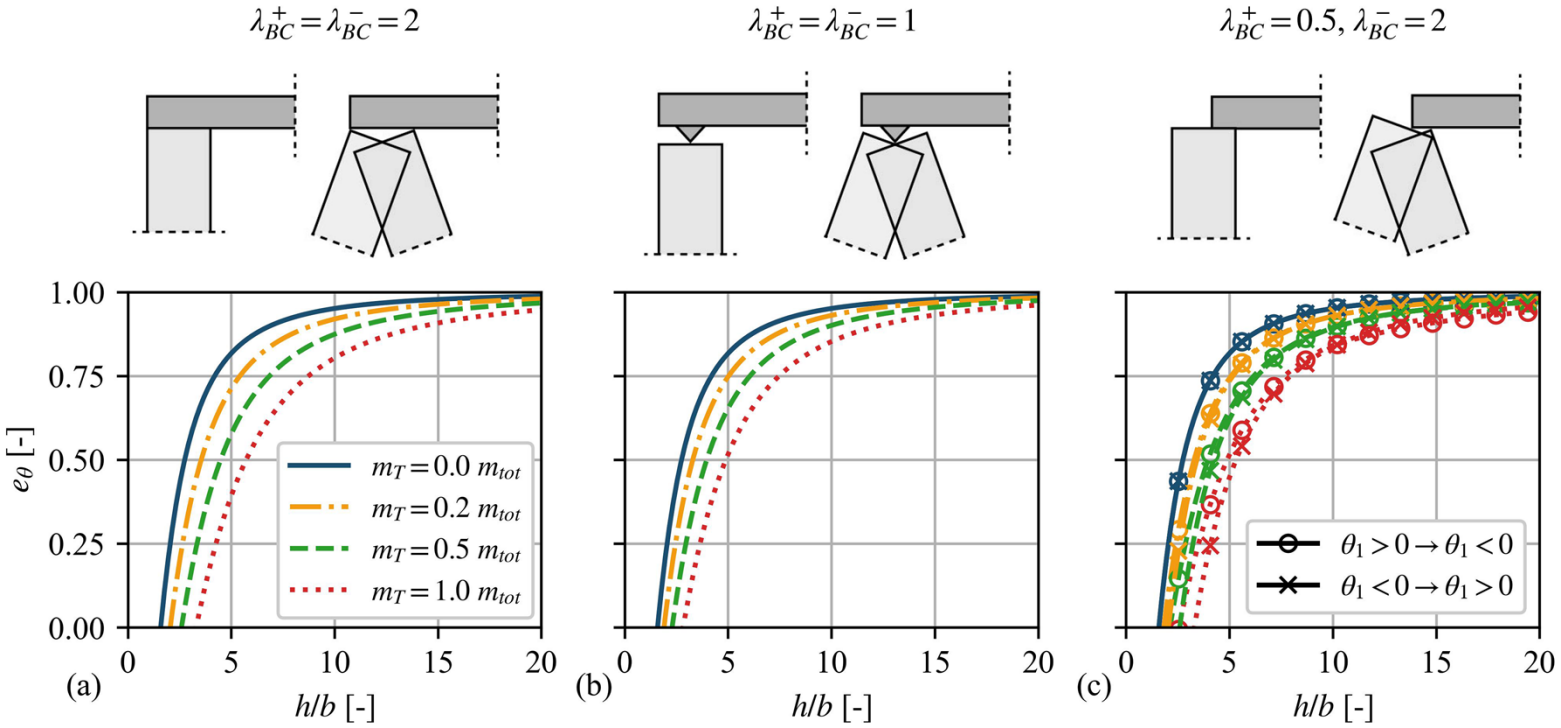
Angular coefficient of restitution $e_{\theta }$ for different wall aspect ratios and overburden mass, assuming a fixed hinge height ${h_{1} / h} = 0.7$ and alternative boundary conditions: **a** “clamped”, **b** “pinned”, and **c** asymmetric

To illustrate the behaviour of the analytical angular CoR, Fig. 5 plots $e_{\theta }$ for a range of aspect ratios (slenderness) of VSSW masonry structures. The plot shows four representative values of the top mass $m_{T}$ corresponding to 0, 0.2, 0.5 and 1.0 of the total mass of the wall, including all three BCs shown in Fig. 2**d**–**f**, while fixing the hinge height at ${h_{1} / h} = 0.7$ for all cases. It is worth highlighting that ${m_{T} }/ {m_{tot}} = 0.2$ corresponds approximately to the weight of an overlaying diaphragm in the case of a single-storey masonry building [16], ${m_{T} }/ {m_{tot}} = 0.5$ corresponds to a parapet resting on the top of the wall together with an overlaying diaphragm, and ${m_{T} }/ {m_{tot}} = 1$ corresponds to an overlaying storey above the VSSW with the same height. In general, Fig. 5 shows that stockier VSSW walls dissipate more energy, in accordance with the case of a single rocking block [35]. Importantly, both the mass on top $m_{T}$ and the BCs have an influence on the energy dissipation of the VSSW. More specifically, the presence of a higher mass on top results in a reduction of the angular CoR and, thus, an increase in the energy dissipation for all examined BCs. Moreover, Fig. 5 indicates that the “pinned” BC (Fig. 2**e**) dissipates less energy than the “clamped” configuration (Fig. 2**d**), especially for larger values of $m_{T}$. Furthermore, the angular CoR of the asymmetric BCs (Fig. 2**f**) is characterised by different values of $e_{\theta }$, depending on the direction of impact, especially for higher values of $m_{T}$. Finally, Fig. 5 implicitly illustrates the importance of the top mass $m_{T}$ compared to the overload ${\rm N}$ on the angular CoR $e_{\theta }$, since ${\rm N}$ does not affect $e_{\theta }$ and thus corresponds to the case of zero top mass in Fig. 5 (solid blue line). Overall, Fig. 5 illustrates that both the mass on top and the BCs have a noteworthy influence on the energy dissipation of the VSSW, even in the case where the hinge height remains the same before and after impact.

### Extraction of the angular coefficient of restitution from the response-history

The quantification of the angular CoR after an experimental campaign may be done following the original definition of $e_{\theta }$, i.e. by measuring the angular velocities of the VSSW right before and after each impact. Despite the seemingly straightforward procedure of this approach, two main reasons make the experimental acquisition of the angular velocities a challenging task [20, 34, 38–41]. Firstly, the time-instants that an impact “starts” and “ends” cannot be defined objectively, as the impact phenomenon is physically continuous and not instantaneous, as assumed by the phenomenological definition of the angular CoR [35]. In addition, during impacts, the velocity of the structure increases at the time-instants of impact. Thus, accurate acquisition of the velocities requires a high sampling rate, which is not always experimentally feasible. Therefore, the need for a simpler approach to extract the angular CoR after an experimental campaign is evident. To this end, one may assume that energy is preserved during the pivoting phase (energy is lost only during impacts), employ the maximum rotations of the system ($\theta_{1,\max }$) for the half cycles before and after the impact, and compute the associated energy loss and the angular CoR $e_{\theta }$. The conservation of energy for each half-cycle reads:

16 $$V_{{\theta_{1,\max } }} - V_{{\theta_{1} = 0}} + \int\limits_{{\theta_{1} = 0}}^{{\theta_{1,\max } }} {\Gamma_{\rm N} d\theta_{1} } = Z_{{\theta_{1} = 0}}$$

Considering the half cycles before and after an impact using Eq. (16), the angular CoR becomes:

17 $$e_{\theta } = \sqrt {\frac{{\left\langle {\frac{{C_{6} }}{{C_{5} }}} \right\rangle^{ + } }}{{\left\langle {\frac{{C_{6} }}{{C_{5} }}} \right\rangle^{ - } }}}$$

where the terms $C_{5}$ and $C_{6}$ are given in Appendix 1. Note that in the case of symmetric BCs, the term $C_{5}$ cancels out from the denominator, and Eq. (17) simplifies to: $e_{\theta } = \sqrt {{{\left\langle {C_{6} } \right\rangle^{ + } } / {\left\langle {C_{6} } \right\rangle^{ - } }}}$. The use of Eq. (17) is illustrated in Sect. 4.1.

## Influence of the overburden mass and boundary conditions on the dynamic response of the VSSW

With the aim of giving insights into the influence of the overburden mass and boundary conditions (BCs) on the response of the VSSW, this Section presents a series of comparative dynamic analyses. The investigation starts with the free-rocking response of one prototypical wall, assuming a variety of BCs, overburden mass and load scenarios. Afterwards, the same wall is examined under sine pulse excitations, and the response is compared in terms of overturning spectra. Finally, an extensive Incremental Dynamic Analysis (IDA) investigation of nine different walls subjected to 30 ground motion records allows for a more holistic appraisal of the importance of the overburden mass and boundary conditions on the VSSW.

### Free-rocking

**Fig. 6:**
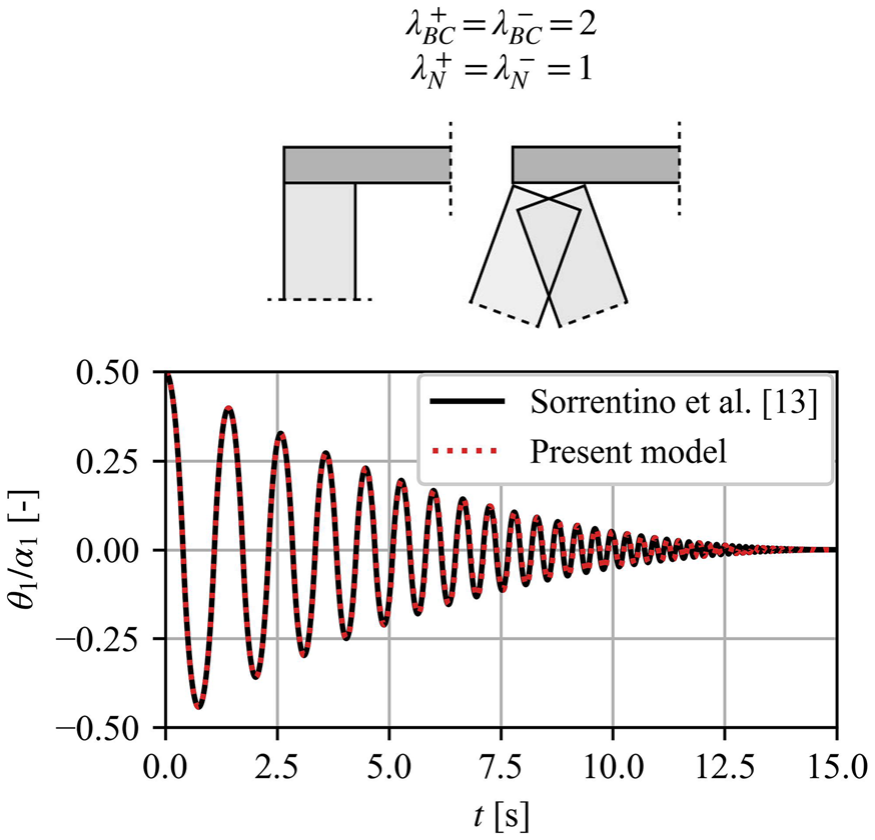
Comparison of free-rocking response between the present and the Sorrentino et al. [13] model. Vertical spanning strip wall details: $2h = 6{\text{ [m]}}$, $2b = 0.5{\text{ [m]}}$, $N = 0.5W$, $\lambda_{BC} = 2$, $\lambda_{N} = 1$ and $m_{T} = 0$

Figure 6 illustrates the free-rocking response of a representative VSSW with a total height of $2h = 6{\text{ [m]}}$, width $2b = 0.5{\text{ [m]}}$ and overload $N = 0.5W$, adopting the model proposed by Sorrentino et al. [13] and the present model. To reproduce the BCs and overload position in [13], we set $\lambda_{BC} = 2$, $\lambda_{N} = 1$ and $m_{T} = 0$. The intermediate hinge height of the present model is computed by minimizing Eq. (11), similar to Sorrentino et al. [13]. Figure 6 illustrates the direct equivalence between the two models. More specifically, by setting $\lambda_{BC} = 2$, $\lambda_{N} = 1$ and $m_{T} = 0$ in the present model, the EOM and the angular CoR (Eqs. (9), (10) and (13)) align closely with those in Sorrentino et al. [13] (Eqs. (10), (11) and (19) in [13]). The only difference lies in that Sorrentino et al. [13] adopted a linearised relation between $\theta_{1}$ and $\theta_{2}$ (Eqs. (2–4) in [13]), whereas the present model preserves the nonlinearity of the system (Eqs. (1) and (3)). As Fig. 6 shows, this linearisation has negligible influence on practical values of the slenderness ($\alpha_{1}$ and $\alpha_{2}$) and rotation angles ($\theta_{1}$ and $\theta_{2}$) [13]. Nevertheless, the plurality of the different BCs and the inclusion of overburden mass that the present model accommodates offer a novel and generalised formulation capable of simulating broader real-world conditions.

**Fig. 7:**
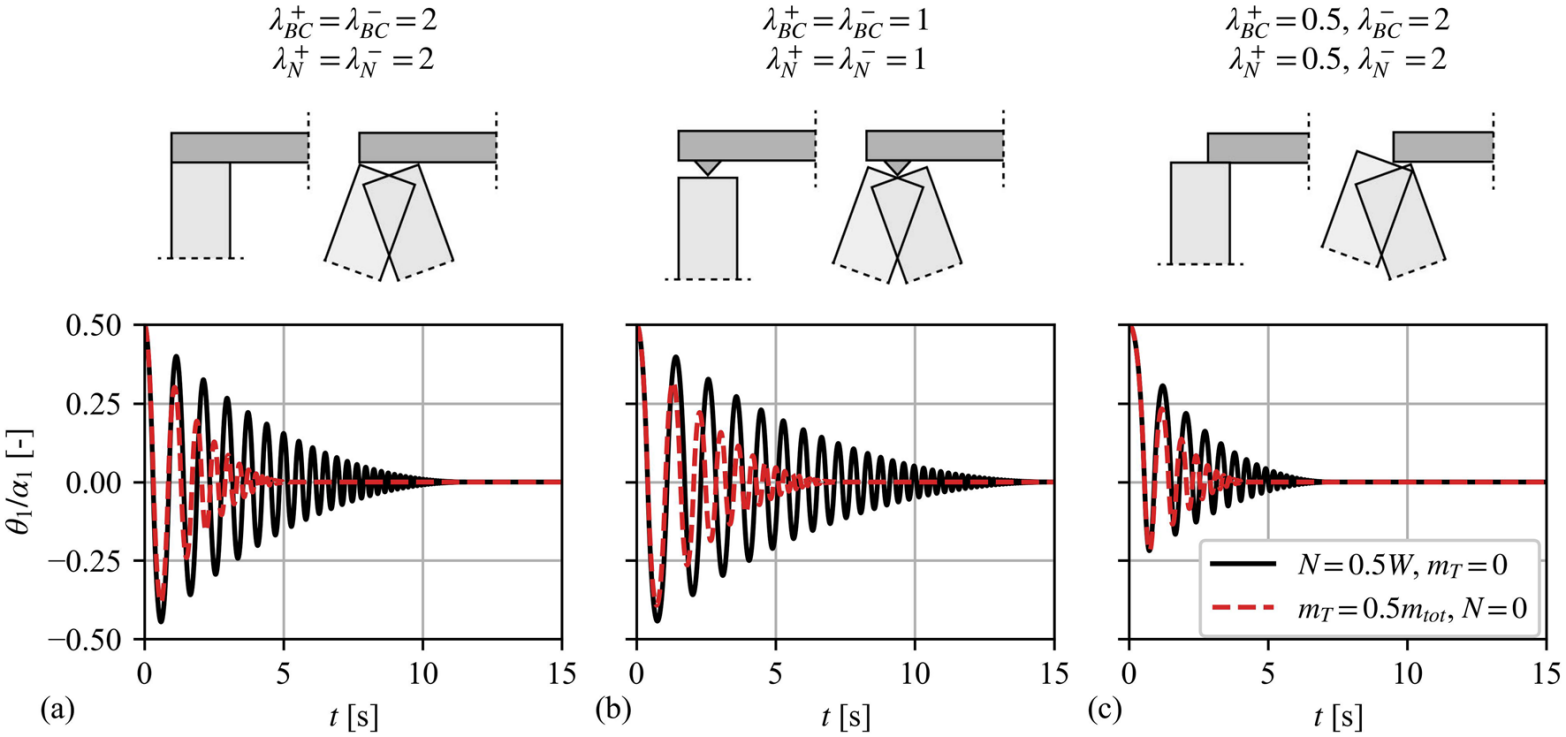
Comparison of free-rocking response among different boundary conditions: **a** “clamped”, **b** “pinned”, **c** asymmetric, and different overburden scenarios: overload $N = 0.5W$ (solid black line), and mass on top $m_{T} = 0.5m_{tot}$ (dashed red line). Vertical spanning strip wall details: $2h = 6{\text{ [m]}}$ and $2b = 0.5{\text{ [m]}}$

Figure 7 adopts the same wall configuration of Fig. 6 ($2h = 6{\text{ [m]}}$, $2b = 0.5{\text{ [m]}}$) and highlights the influence the different BCs, overburden mass, and load scenarios have on the free-rocking response of the wall. More specifically, Fig. 7 compares the response of a wall with an overload $N = 0.5W$ (solid black line) with that of a wall with an equivalent mass on top $m_{T} = 0.5m_{tot}$ (dashed red line). Furthermore, Fig. 7**a** accounts for the “clamped” configuration of Fig. 2**d**, Fig. 7**b** examines the “pinned” configuration of Fig. 2**e**, and Fig. 7**c** replicates the asymmetric configuration of Fig. 2f. Note that the hinge height of each case and for each direction is computed by minimising Eq. (11), while the angular CoR is computed using Eq. (13) considering also that the condition of Eq. (14) is always satisfied. Figure 7 indicates that the presence of the overburden mass $m_{T}$ results in a significantly higher energy dissipation compared to the equivalent overload cases. This stems from the influence of the top mass on the angular CoR (Eq. (13)). Moreover, Fig. 7 shows that the “pinned” BCs (Fig. 7**b**) are the least dissipative, while, as expected, the asymmetric BCs present markedly asymmetric responses. Overall, Fig. 7 highlights the influence of both the overburden mass and different BCs on the free-rocking response of the examined VSSW.

**Fig. 8:**
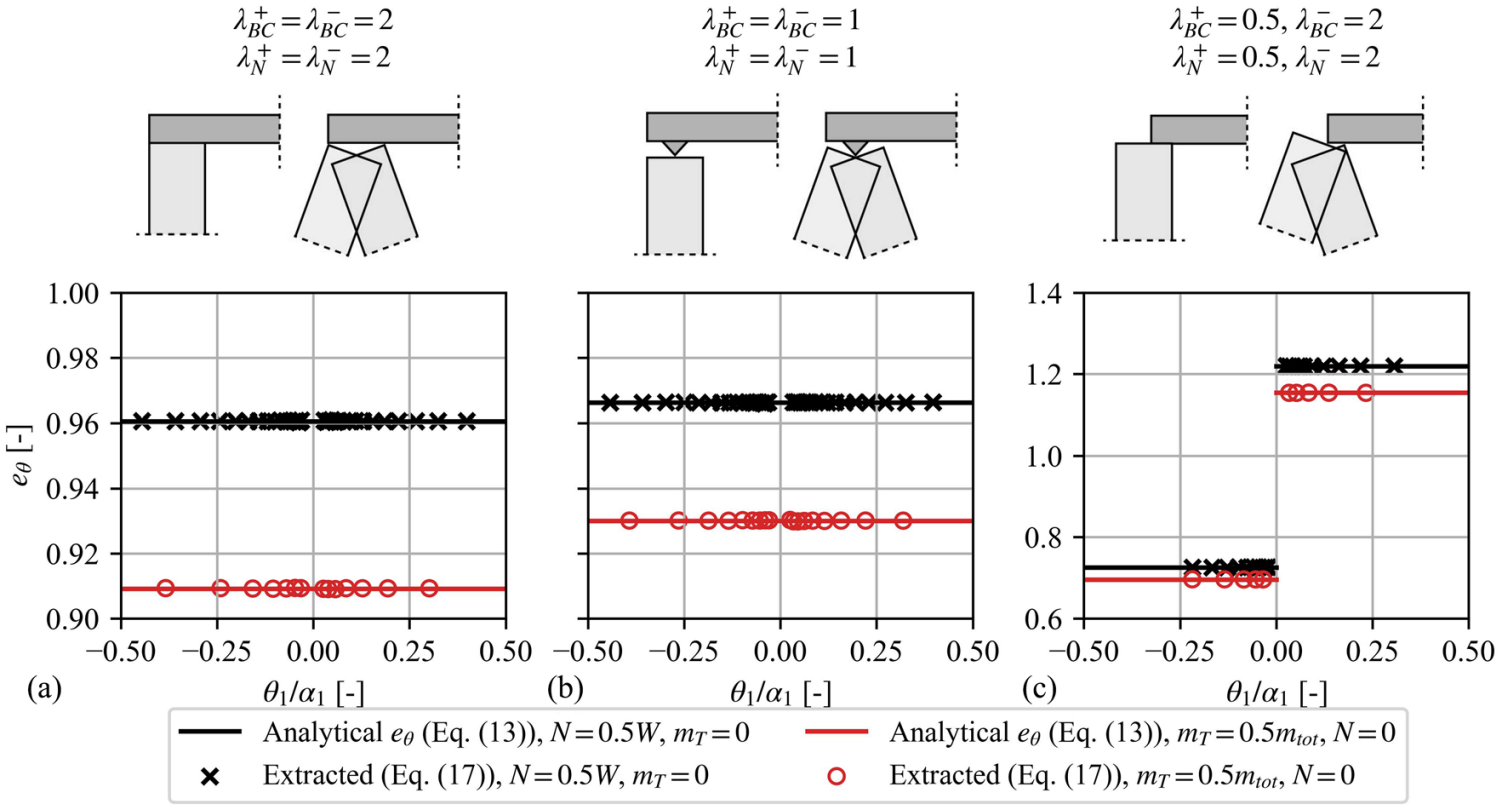
Extraction of the angular coefficient of restitution $e_{\theta }$ of the free-rocking response shown in Fig. 8 with the use of Eq. (17)

In continuation of Fig. 7, Fig. 8 validates the use of Eq. (17) for extracting the assumingly unknown angular CoR from the response-history of Fig. 7. In particular, Fig. 8 together with Eq. (17) employ solely the geometrical properties of the analysed VSSWs and the peak amplitudes of the responses of Fig. 7 to extract $e_{\theta }$. This becomes extremely useful for the case of an experimental campaign, where the angular CoR is unknown and is extracted by the measured response. Figure 8 validates Eq. (17) showing that the extracted angular CoR of Eq. (17) matches perfectly the analytical angular CoR of Eq. (13) used in Fig. 7. Indeed, Fig. 8 shows that Eq. (17) is able to predict correctly $e_{\theta }$ for all the examined cases, including the most demanding asymmetric BCs. It is worth noting that in Fig. 8**c**, higher than unity values of $e_{\theta }$ are due to the asymmetry of the BCs and do not reflect an increase in the system’s energy, as this is restrained by Eqs. (14) and (15). This behaviour stems from the different inertia of the system for positive (counter-clockwise) and negative (clockwise) rotations, which is represented by the term $C_{5}$ in Eqs. (14) and (15). More specifically, a lower value of $\lambda_{BC}^{ + }$ (or $\lambda_{N}^{ + }$) in comparison with $\lambda_{BC}^{ - }$ (or $\lambda_{N}^{ - }$) results in a higher hinge height (as shown in Fig. 3a), which corresponds to a higher value of the inertia of the system (term $C_{5}$). As a result, during an impact, from a positive pre-impact to a negative post-impact rotation of the bottom block, the system experiences a reduction in its inertia ($C_{5}$), thus allowing an increase in angular velocity without an increase in energy.

### Overturning spectra under sinusoidal pulses

Previous studies on the response of the single rocking block have considered mathematical pulses [35, 42–44] as representatives of pulse-like ground motions [45–47]. Common examples of such pulses include sine and cosine functions, symmetric or asymmetric Ricker wavelets, among others [48]. In light of the above, this Section constructs the overturning spectra of the previously examined VSSW of Fig. 7 when subjected to a series of sinusoidal ground accelerations.

**Fig. 9:**
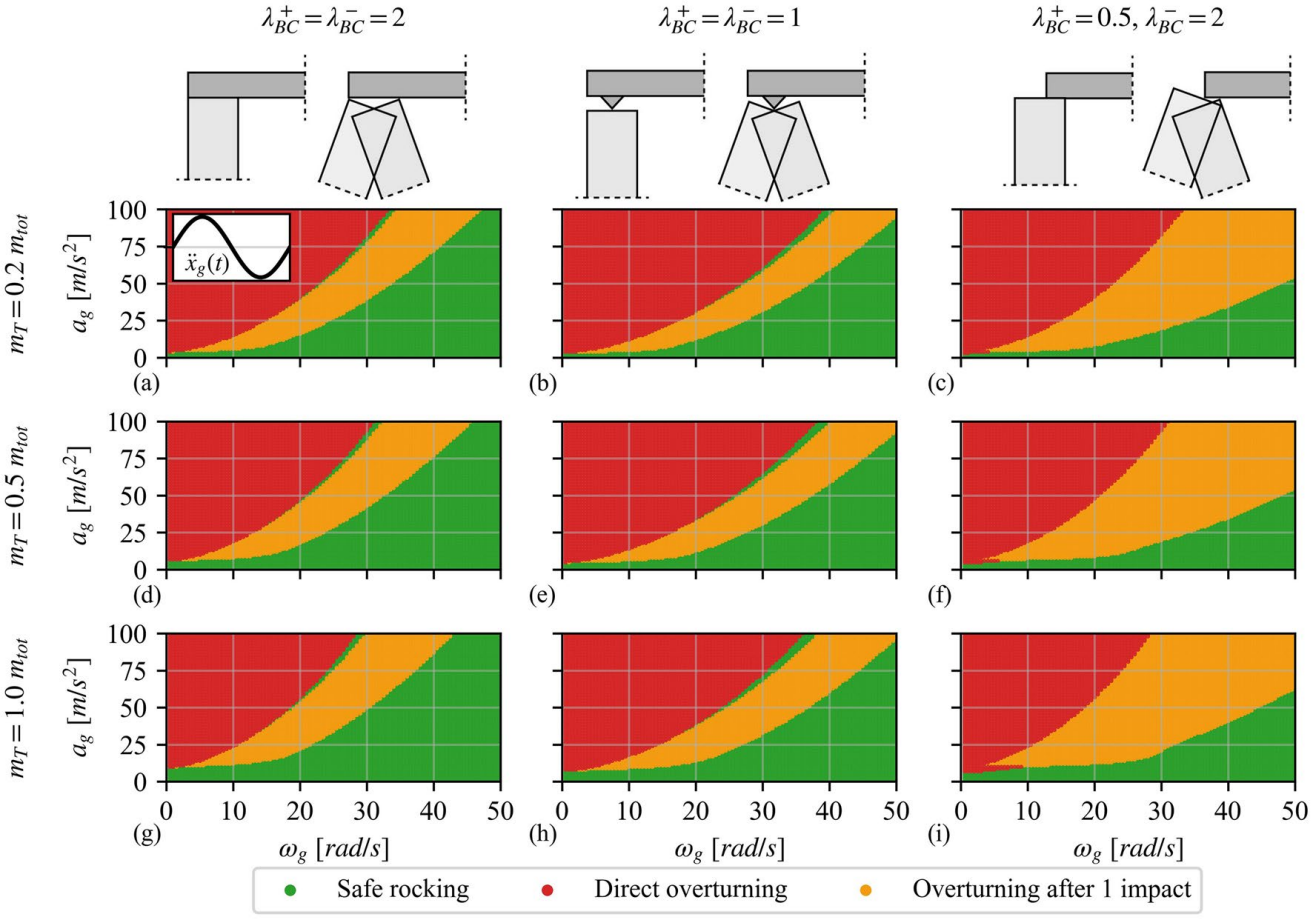
Overturning spectra of a VSSW for different overburden mass: **a**–**c** $m_{T} = 0.2m_{tot}$, **d**–**f** $m_{T} = 0.5m_{tot}$ and (**g**–**i**) $m_{T} = 1.0m_{tot}$, and boundary conditions: **a**, **d**, **g** “clamped”, **b**, **e**, **c** “pinned”, and **c**, **f**, **i** asymmetric. Vertical spanning strip wall details: $2h = 6{\text{ [m]}}$, $2b = 0.5{\text{ [m]}}$ and $N = 0$

Figure 9 shows the overturning spectra of a VSSW with $2h = 6{\text{ [m]}}$ and $2b = 0.5{\text{ [m]}}$ when subjected to sine pulse excitations of various acceleration amplitudes $a_{g}$ and angular frequencies $\omega_{g}$, considering three different values of the normalised overburden mass (i.e. $m_{T} = 0.2m_{tot}$ Fig. 9**a**–**c**, $m_{T} = 0.5m_{tot}$ Fig. 9**d**–**f** and $m_{T} = 1.0m_{tot}$ Fig. 9**g**–**i**) and three BCs (i.e. “clamped” Fig. 9**a**, **d**, **g**, “pinned” Fig. 9**b**, **e**, **h** and asymmetric Fig. 9**c**, **f**, **i**). The horizontal axis reflects the angular frequency $\omega_{g}$ while the vertical axis refers to the acceleration amplitude $a_{g}$ of the pulse. Each spectrum is constructed for a grid of 100 × 100 points, illustrating the results of 10 000 analyses. Figure 9 reports the results of the analyses using colour distinction: green dots represent safe rocking, red dots indicate direct overturning (without prior impact), and orange dots illustrate overturning after one impact. From a numerical perspective, overturning is assumed when ${\theta_{1}}/ {\alpha_{1} }  \ge 2$, which is at least two times more than the instability angle $\theta_{1,inst}$ (Fig. 3b). First of all, it is worth noting that the overturning spectra of Fig. 9 have a close resemblance with that of a single rocking block [43], briefly summarised as follows: (i) higher amplitudes are required to cause overturning for higher frequencies of excitation, (ii) there are two regions of collapse associated with direct overturning (without prior impact) or overturning after one impact, and (iii) in between those regions of collapse there are regions of safe rocking. Nonetheless, this region of safe rocking is not present in the case of asymmetric BC (Fig. 9**c**, **f**, **i**). In addition, Fig. 9 shows that an increase of the overburden mass $m_{T}$ slightly enhances the seismic stability of the VSSW as it shrinks the overturning area (see e.g. Figure 9**a** versus Fig. 9**g**). As a consequence, the aforementioned stability enhancement results in new safe regions, while previous safe regions become unsafe. For example, for the “clamped” BCs (Fig. 9**a**), the examined VSSW with $m_{T} = 0.2m_{tot}$ survives a sinusoidal excitation with $\omega_{g} = 30{\text{ [rad/s]}}$ and $a_{g} = 80\;{ [}{{\text{m}} / {{\text{s}}^{{2}} }}{]}$, while the same VSSW configuration with $m_{T} = 0.5m_{tot}$ (Fig. 9**d**) or $m_{T} = 1.0m_{tot}$ (Fig. 9**g**), under the same excitation, overturns after one impact. This indicates that ignoring the presence of the overburden mass is not always conservative, as the change in the dynamics of the system might be either beneficial or detrimental to the seismic stability of the structure. Furthermore, in general, the asymmetric BCs (Fig. 9**c**, **f**, **i**) appear to be notably more vulnerable than the “pinned” BCs (Fig. 9**b**, **e**, **h**), which is in turn more vulnerable than the “clamped” BCs (Fig. 9**a**, **d**, **g**), as the safe region is considerably shrunk. In particular, the greater vulnerability of the asymmetric BCs arises from the expanded region of overturning after one impact, which is linked to the overturning toward the weaker side of the VSSW (i.e. with $\lambda_{BC}^{ + } = 0.5$).

**Fig. 10:**
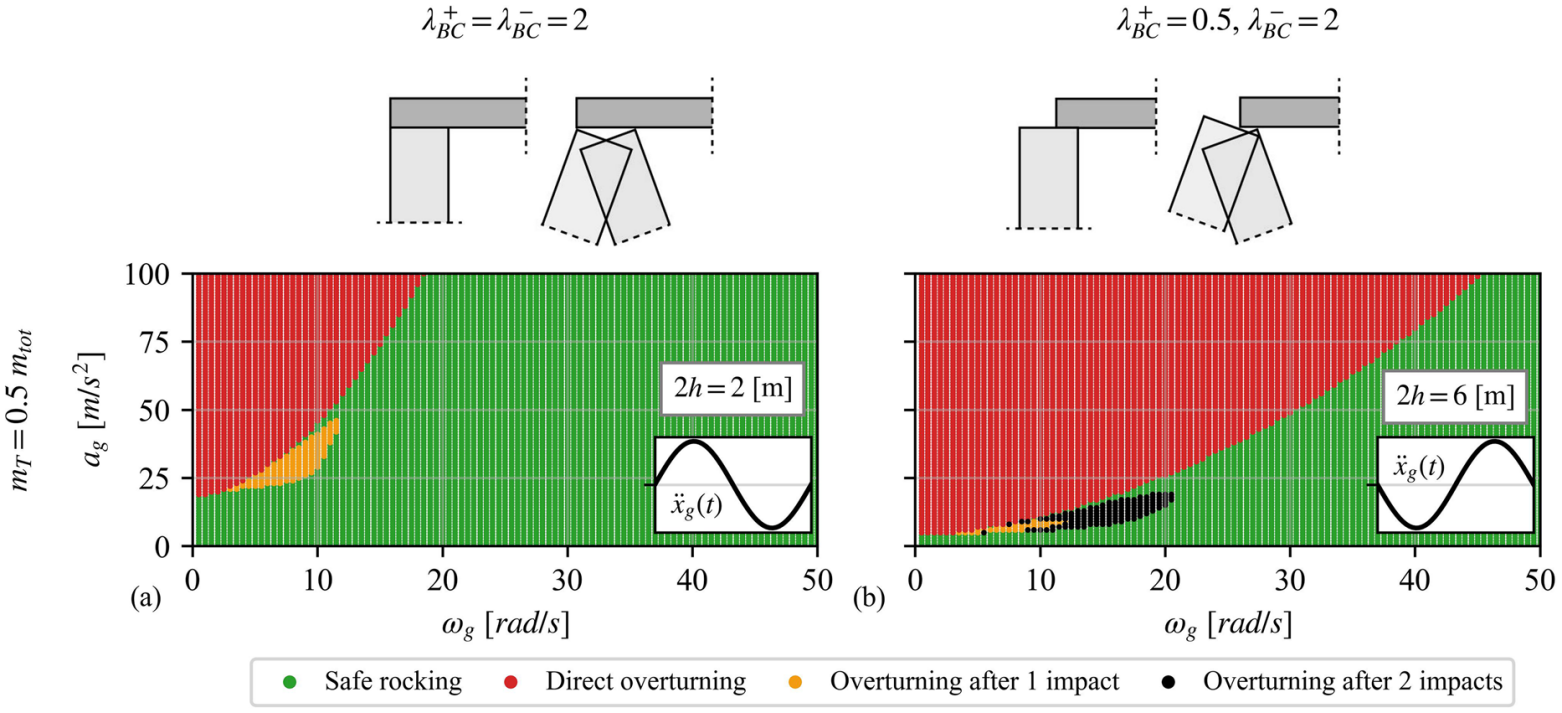
Overturning spectra of a VSSW with **a** $2h = 2{\text{ [m]}}$, $2b = 0.5{\text{ [m]}}$, $m_{T} = 0.5m_{tot}$, $N = 0$, “clamped” boundary conditions under a positive-to-negative sine pulse, and **b** $2h = 6{\text{ [m]}}$, $2b = 0.5{\text{ [m]}}$, $m_{T} = 0.5m_{tot}$, $N = 0$, asymmetric boundary conditions under a negative-to-positive sine pulse

Notably, the overturning spectra of Fig. 9 do not completely resemble the pertinent spectrum of a rocking block due to the absence of the “bubble”-shaped overturning after one impact area. This discrepancy may be due to the geometry of the wall (e.g. slenderness, size), the directivity of the ground motion, the boundary conditions, the value of the angular CoR, or a combination of the above. Figure 10 illustrates cases where the overturning after one impact area is substantially shrunk (compared to Fig. 9), and the spectrum becomes visually similar to that of a rocking block. Specifically, Fig. 10**a** plots the overturning spectrum of a VSSW with base width $2b = 0.5{\text{ [m]}}$, height $2h = 2{\text{ [m]}}$, “clamped” BCs (Fig. 2**d**) and $m_{T} = 0.5m_{tot}$. Figure 10**a** highlights the influence of the size and slenderness on the stability of the VSSW. It becomes apparent that the overall seismic performance of the VSSW is dictated by the geometry of the structure as, contrary to Fig. 9**d**, the structure of Fig. 10**a** is more stable with both overturning modes (i.e. without prior impact and after one impact) being substantially shrunk. A similar trend is observed when the ground excitation changes direction. Figure 10b illustrates the overturning spectrum of a VSSW with base width $2b = 0.5{\text{ [m]}}$, height $2h = 6{\text{ [m]}}$, asymmetric BCs (Fig. 2**f**) and $m_{T} = 0.5m_{tot}$, considering opposite direction of the sinusoidal pulse (compared to Fig. 9**f**). Again, the safe region of the VSSW is significantly increased compared to Fig. 9**f**. In addition to the expansion of the safe region, Fig. 10**b** also shows the emergence of a new region where overturning occurs after two impacts. Therefore, it becomes clear that even though a VSSW and a block share some similarities, they also have some important discrepancies due to their different complexity, which has an impact on their dynamic behaviour. Overall, Fig. 9 and Fig. 10 highlight the importance of the overburden mass, BCs, geometrical characteristics, ground motion directivity, among others, when assessing the seismic stability of a VSSW.

### Overturning fragility curves under recorded earthquakes

This Section extends the previous comparisons to recorded ground motion excitations. Importantly, the highly nonlinear behaviour of the present model together with the high record-to-record variability provide only a partial view in the case of deterministic comparisons. To this end, this study employs a suite of 30 ordinary ground motion records and investigates 9 different VSSWs. For each wall, both the overburden mass and the BCs are varied, and Incremental Dynamic Analysis (IDA) [49] are performed. Finally, the comparison is conducted in terms of overturning empirical fragility curves.

The adopted ground motions are characterised by large magnitudes (M_w_ = 6.5–6.9) and moderate rupture distances ($R_{rup} < 32.6{\text{ km}}$) [50]. The characteristics of the adopted ground motions are listed in Table 1 of the Appendix 2. Nine cases are considered regarding the geometry of the VSSWs, covering common structural configurations: the width $2b$ is equal to 0.35, 0.5 and 0.7 [m], while the aspect ratio ${h /b}$ is equal to 9, 12 and 15 [16]. Furthermore, since the comparison concerns the overburden mass and BCs, the “clamped” BCs with $m_{T} = 0.5m_{tot}$ is assumed as a reference. This study investigates variations of the normalised overburden mass ($m_{T} = 0.2m_{tot}$ and $m_{T} = 1.0m_{tot}$ instead of $m_{T} = 0.5m_{tot}$), overload ($N = 0.5W$ instead of mass), and BCs (“pinned” and asymmetric instead of “clamped”). Subsequently, for each case, IDAs are conducted for all the 30 ground motions of Table 1 and the empirical probability of overturning is computed with respect to the Peak Ground Velocity (PGV) [51, 52]. More specifically, each IDA is constructed with a constant PGV scaling step of $0.015{\text{ [m/s]}}$ and is terminated when the first overturning occurs (i.e. possible “resurrections” [53] are neglected). Similar to Sect. 4.2, overturning is assumed when ${{\theta_{1}} / {\alpha_{1} }} \ge 2$.

**Fig. 11:**
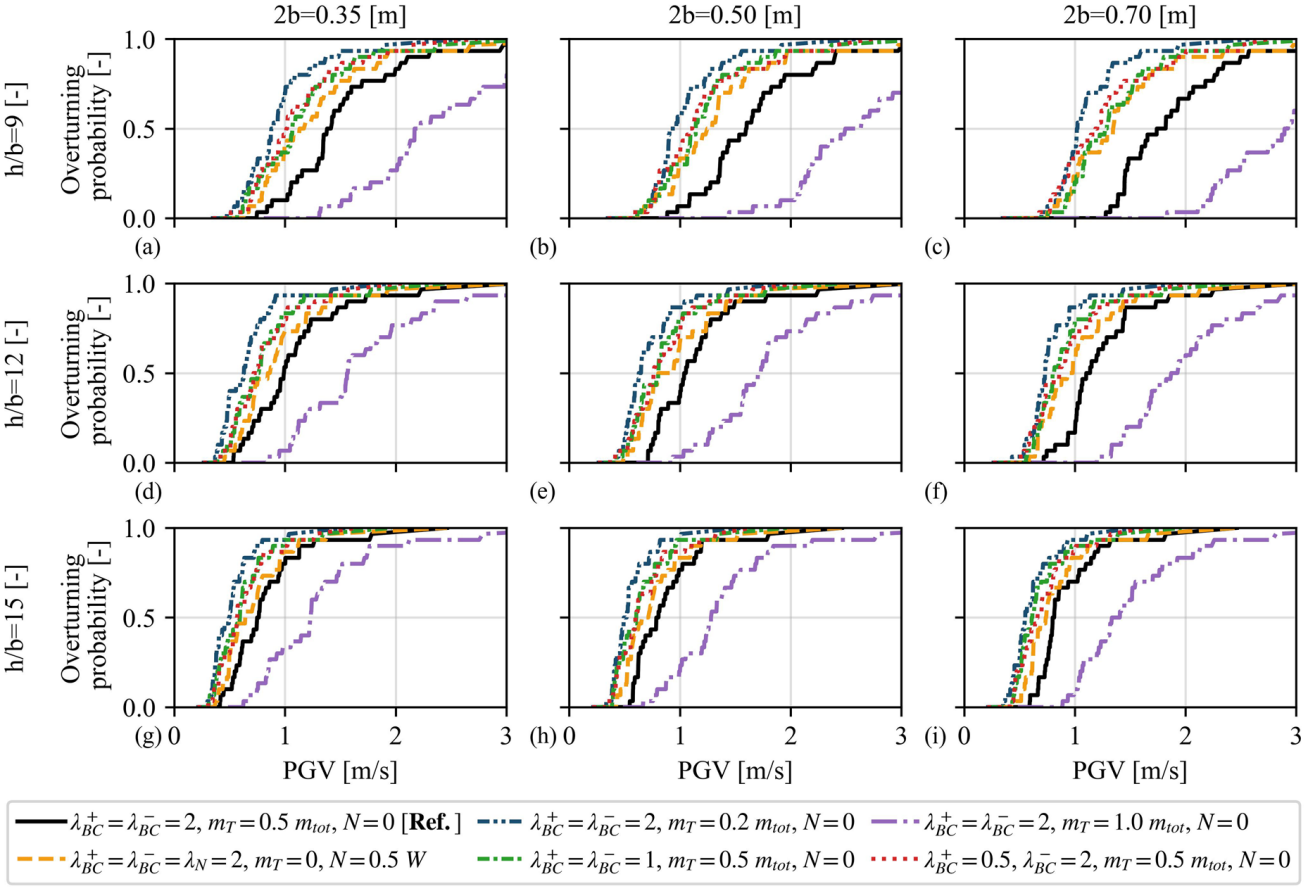
Overturning fragility curves of vertical spanning strip walls with base width **a**, **d**, **g** 0.35, **b**, **e**, **h** 0.5, and **c**, **f**, **i** 0.7 [m], aspect ratio ${h / b}$ **a**–**c** 12, **d**-**f** 12, and **g**–**i** 15, for different overburden and boundary condition scenarios

Figure 11 summarises the results of 1 620 IDAs (which correspond to 17 657 dynamic analyses, in total) in terms of empirical overturning fragility curves. As expected, Fig. 11 illustrates that stockier VSSW configurations are less vulnerable to overturning (see e.g. Figure 11**a** versus Fig. 11**c** or Fig. 11**a** versus Fig. 11**g**). Furthermore, Fig. 11 depicts that increasing the mass on top $m_{T}$ leads to more stable configurations with lower overturning probabilities (e.g. dashdot purple lines versus densely dashdotdotted blue lines). Moreover, a notable difference in the overturning fragility is observed when an overburden mass (solid black lines) is considered instead of an overload (dashed orange lines), despite having the same normalised value of 0.5. The latter case displays higher vulnerability to overturning. In addition, Fig. 11 indicates that the different BCs influence significantly the overturning probability, with the “clamped” case (solid black lines) outperforming the “pinned” (densely dashdotted green lines) and asymmetric (dotted red lines) cases. Interestingly, the last two cases show marginal differences for stockier walls (Fig. 11**a**–**c**). In sum, Fig. 11 provides a holistic overview of the response of the VSSW model subjected to recorded ground motions and highlights the importance of both the overburden mass and BCs on its seismic vulnerability.

## Conclusions

This paper presents a numerical model for the analysis of a vertical spanning strip wall based on rocking dynamics. This work extends current knowledge by incorporating the positional parametrisation of the boundary conditions at the top of the wall and accounting for the presence of the overburden mass. These extensions enable a more accurate representation of a wide variety of boundary conditions and overburden scenarios found in realistic unreinforced masonry structures. Importantly, this study demonstrates the significance of these parameters on the response of the vertical spanning strip wall through a series of comparative analyses.

The nonlinear equation of motion is derived using Lagrangian dynamics, followed by the minimum ground acceleration capable of initiating rocking motion, the estimation of the expected hinge height, and the computation of the instability angle. It is shown that the last two are decidedly influenced by the boundary conditions on top of the wall.

The energy losses during the impacts of the vertical spanning strip wall are also examined, and two methods are proposed for quantifying the angular coefficient of restitution. The first method provides an analytical estimation of the coefficient of restitution, useful for predictive applications. To this end, impulsive dynamics are employed and, within postulated assumptions, a formula for the angular coefficient of restitution is derived. This demonstrates the dependence of the angular coefficient of restitution on the overburden mass and the boundary conditions at the top of the vertical spanning strip wall. The second method facilitates the extraction of the angular coefficient of restitution from a given free-rocking time-history response assuming that energy is lost solely during impacts. This method is particularly useful for the characterisation and calibration of the energy losses after an experimental campaign, while its validity and use are demonstrated for controlled numerical tests.

Finally, the work highlights various aspects of the proposed model using comparative analyses. These include free- and forced-rocking simulations under sinusoidal pulse excitations and real ground motion records. More specifically, the free-rocking comparisons demonstrate the effect of an overburden mass and boundary conditions on the energy dissipation of a representative vertical spanning strip wall. Furthermore, the analyses of the same wall under sine pulse excitations demonstrate that variations in the overburden mass and boundary conditions alter the system’s dynamics and, therefore, influence the corresponding safe and overturning regions. Importantly, the directivity of the sine pulse appears to be detrimental in the case of asymmetric boundary conditions. Ultimately, the most comprehensive view of the vertical spanning strip wall’s vulnerability is attained through a series of real ground motion forced-rocking analyses. Therein, empirical fragility curves highlight the importance of the overburden mass and boundary conditions on the seismic response of nine different walls.

## Appendix 1 Supplementary terms of Sects. 2 and 3

This Section presents the supplementary terms used in Sects. 2 and 3.

18 $$C_{1} = {\text{sgn}} \theta_{1} \cos \varphi \tan \alpha_{2} \left[ {\frac{{\sin \left( {\alpha_{1} - \left| {\theta_{1} } \right|} \right)}}{{\sin \alpha_{1} }} + \frac{{\lambda_{BC} }}{2} - 1} \right]$$

19 $$\frac{{dC_{1} }}{{d\theta_{1} }} = - \cos \varphi \tan \alpha_{2} \frac{{\cos \left( {\alpha_{1} - \left| {\theta_{1} } \right|} \right)}}{{\sin \alpha_{1} }}$$

20 $$\frac{{d^{2} C_{1} }}{{d\theta_{1}^{2} }} = - {\text{sgn}} \theta_{1} \cos \varphi \tan \alpha_{2} \frac{{\sin \left( {\alpha_{1} - \left| {\theta_{1} } \right|} \right)}}{{\sin \alpha_{1} }}$$

21 $$C_{2} = - 2\cos \left( {\alpha_{1} - \left| {\theta_{1} } \right|} \right) - \frac{{\sin \alpha_{1} }}{{\sin \alpha_{2} }}\frac{{dC_{1} }}{{d\theta_{1} }}\frac{1}{{\sqrt {1 - C_{1}^{2} } }}\cos \left( {\alpha_{2} - \left| {\theta_{2} } \right|} \right)$$

22 $$\frac{{dC_{2} }}{{d\theta_{1} }} = - 2{\text{sgn}} \theta_{1} \sin \left( {\alpha_{1} - \left| {\theta_{1} } \right|} \right) - \frac{{\sin \alpha_{1} }}{{\sin \alpha_{2} \left( {1 - C_{1}^{2} } \right)}}\left\{ \begin{gathered} \left[ {\frac{{d^{2} C_{1} }}{{d\theta_{1}^{2} }}\sqrt {1 - C_{1}^{2} } + \left( {\frac{{dC_{1} }}{{d\theta_{1} }}} \right)^{2} \frac{{C_{1} }}{{\sqrt {1 - C_{1}^{2} } }}} \right]\cos \left( {\alpha_{2} - \left| {\theta_{2} } \right|} \right) \hfill \\ - {\text{sgn}} \theta_{1} \left( {\frac{{dC_{1} }}{{d\theta_{1} }}} \right)^{2} \sin \left( {\alpha_{2} - \left| {\theta_{2} } \right|} \right) \hfill \\ \end{gathered} \right\}$$

23 $$C_{3} = {\text{sgn}} \theta_{1} \left[ {2\sin \left( {\alpha_{1} - \left| {\theta_{1} } \right|} \right) - \frac{{\sin \alpha_{1} }}{{\sin \alpha_{2} }}\frac{{dC_{1} }}{{d\theta_{1} }}\frac{1}{{\sqrt {1 - C_{1}^{2} } }}\sin \left( {\alpha_{2} - \left| {\theta_{2} } \right|} \right)} \right]$$

24 $$\frac{{dC_{3} }}{{d\theta_{1} }} = - 2\cos \left( {\alpha_{1} - \left| {\theta_{1} } \right|} \right) - {\text{sgn}} \theta_{1} \frac{{\sin \alpha_{1} }}{{\sin \alpha_{2} \left( {1 - C_{1}^{2} } \right)}}\left\{ \begin{gathered} \left[ {\frac{{d^{2} C_{1} }}{{d\theta_{1}^{2} }}\sqrt {1 - C_{1}^{2} } + \left( {\frac{{dC_{1} }}{{d\theta_{1} }}} \right)^{2} \frac{{C_{1} }}{{\sqrt {1 - C_{1}^{2} } }}} \right]\sin \left( {\alpha_{2} - \left| {\theta_{2} } \right|} \right) \hfill \\ + {\text{sgn}} \theta_{1} \left( {\frac{{dC_{1} }}{{d\theta_{1} }}} \right)^{2} \cos \left( {\alpha_{2} - \left| {\theta_{2} } \right|} \right) \hfill \\ \end{gathered} \right\}$$

25 $$C_{4} = 2{\text{sgn}} \theta_{1} \sin \left( {\alpha_{1} - \left| {\theta_{1} } \right|} \right) - 2\frac{{\sin \alpha_{1} }}{{\tan \alpha_{2} }}\frac{{dC_{1} }}{{d\theta_{1} }}\frac{1}{{\sqrt {1 - C_{1}^{2} } }}\frac{{\sin \left( {\varphi + \theta_{2} } \right)}}{\cos \varphi }$$

26 $$\frac{{dC_{4} }}{{d\theta_{1} }} = - 2\cos \left( {\alpha_{1} - \left| {\theta_{1} } \right|} \right) - 2\frac{{\sin \alpha_{1} }}{{\tan \alpha_{2} \left( {1 - C_{1}^{2} } \right)}}\left\{ \begin{gathered} \left[ {\frac{{d^{2} C_{1} }}{{d\theta_{1}^{2} }}\sqrt {1 - C_{1}^{2} } - \left( {\frac{{dC_{1} }}{{d\theta_{1} }}} \right)^{2} C_{1} } \right]\frac{{\sin \left( {\varphi + \theta_{2} } \right)}}{\cos \varphi } \hfill \\ + \left( {\frac{{dC_{1} }}{{d\theta_{1} }}} \right)^{2} \frac{{\cos \left( {\varphi + \theta_{2} } \right)}}{\cos \varphi } \hfill \\ \end{gathered} \right\}$$

27 $$C_{5} = R_{1}^{2} \left\{ \begin{gathered} m_{1} + m_{2} \left[ {\cos^{2} \alpha_{1} + \sin^{2} \alpha_{1} \left( {2 + \frac{{\tan \alpha_{2} }}{{\tan \alpha_{1} }}} \right)^{2} } \right] \hfill \\ + m_{T} \sin^{2} \alpha_{1} \left( {2 + \lambda_{BC} \frac{{\tan \alpha_{2} }}{{\tan \alpha_{1} }}} \right)^{2} \hfill \\ \end{gathered} \right\} + I_{G1} + I_{G2} \left( {\frac{{\tan \alpha_{2} }}{{\tan \alpha_{1} }}} \right)^{2}$$

28 $$C_{6} = \left[ \begin{gathered} gR_{1} \left\{ \begin{gathered} \left( {m_{1} + 2m_{2} + 2m_{T} } \right)\left[ {\cos \left( {\alpha_{1} - \left| {\theta_{1,\max } } \right|} \right) - \cos \alpha_{1} } \right] \hfill \\ + m_{2} \frac{{\sin \alpha_{1} }}{{\sin \alpha_{2} }}\left[ {\cos \left( {\alpha_{2} - \left| {\theta_{2,\max } } \right|} \right) - \cos \alpha_{2} } \right] \hfill \\ + 2m_{T} \frac{{\sin \alpha_{1} }}{{\tan \alpha_{2} \cos \varphi }}\left[ {\sqrt {1 - \left( {\cos \varphi \tan \alpha_{2} \left[ {\frac{{\sin \left( {\alpha_{1} - \left| {\theta_{1,\max } } \right|} \right)}}{{\sin \alpha_{1} }} - 1 + \frac{{\lambda_{BC} }}{2}} \right]} \right)^{2} } - \sqrt {1 - \left( {\frac{{\lambda_{BC} }}{2}\cos \varphi \tan \alpha_{2} } \right)^{2} } } \right] \hfill \\ \end{gathered} \right\} \hfill \\ + NR_{1} \left\{ {2\left[ {\cos \left( {\alpha_{1} - \left| {\theta_{1,\max } } \right|} \right) - \cos \alpha_{1} } \right] + \sin \alpha_{1} \left[ {\lambda_{N} \sin \left( {\left| {\theta_{2,\max } } \right|} \right) + 2\frac{{\cos \left( {\left| {\theta_{2,\max } } \right|} \right) - 1}}{{\tan \alpha_{2} }}} \right]} \right\} \hfill \\ \end{gathered} \right]$$

## Appendix 2 Details of ground motions used in Sect. 4.3

This Section tabulates the characteristics of the ground motions adopted in Sect. 4.3 see Table 1.

**Table 1:** Details of ground motion records of Sect. 4.3, collected from [50]

No	Event	Station	Component [°]	Soil class	Magnitude Mw	Rupture distance [km]	PGA [g]
1	Loma Prieta, 1989	Agnes State Hospital	90	C, D	6.9	28.2	0.159
2	Northridge, 1994	LA, Baldwin Hills	90	B, B	6.7	31.3	0.239
3	Imperial Valley, 1979	Compuertas	285	C, D	6.5	32.6	0.147
4	Imperial Valley, 1979	Plaster City	135	C, D	6.5	31.7	0.057
5	Loma Prieta, 1989	Hollister Diff. Array	255	–, D	6.9	25.8	0.279
6	San Fernando, 1971	LA, Hollywood Stor. Lot	180	C, D	6.6	21.2	0.174
7	Loma Prieta, 1989	Anderson Dam Downstrm	270	B, D	6.9	21.4	0.244
8	Loma Prieta, 1989	Coyote Lake Dam Downstream	285	B, D	6.9	22.3	0.179
9	Imperial Valley, 1979	El Centro Array #12	140	C, D	6.5	18.2	0.143
10	Imperial Valley, 1979	Cucapah	85	C, D	6.5	23.6	0.309
11	Northridge, 1994	LA, Hollywood Storage FF	360	C, D	6.7	25.5	0.358
12	Loma Prieta, 1989	Sunnyvale Colton Ave	270	C, D	6.9	28.8	0.207
13	Loma Prieta, 1989	Anderson Dam Downstrm	360	B, D	6.9	21.4	0.24
14	Imperial Valley, 1979	Chihuahua	12	C, D	6.5	28.7	0.27
15	Imperial Valley, 1979	El Centro Array #13	140	C, D	6.5	21.9	0.117
16	Imperial Valley, 1979	Westmoreland Fire Station	90	C, D	6.5	15.1	0.074
17	Loma Prieta, 1989	Hollister South & Pine	0	–, D	6.9	28.8	0.371
18	Loma Prieta, 1989	Sunnyvale Colton Ave	360	C, D	6.9	28.8	0.209
19	Superstition Hills, 1987	Wildlife Liquefaction Array	90	C, D	6.7	24.4	0.18
20	Imperial Valley, 1979	Chihuahua	282	C, D	6.5	28.7	0.254
21	Imperial Valley, 1979	El Centro Array #13	230	C, D	6.5	21.9	0.139
22	Imperial Valley, 1979	Westmoreland Fire Station	180	C, D	6.5	15.1	0.11
23	Loma Prieta, 1989	Halls Valley	90	C, C	6.9	31.6	0.103
24	Loma Prieta, 1989	WAHO	0	–, D	6.9	16.9	0.37
25	Superstition Hills, 1987	Wildlife Liquefaction Array	360	C, D	6.7	24.4	0.2
26	Imperial Valley, 1979	Compuertas	15	C, D	6.5	32.6	0.186
27	Imperial Valley, 1979	Plaster City	45	C, D	6.5	31.7	0.042
28	Loma Prieta, 1989	Hollister Diff. Array	165	–, D	6.9	25.8	0.269
29	San Fernando, 1971	LA, Hollywood Stor. Lot	90	C, D	6.6	21.2	0.21
30	Loma Prieta, 1989	WAHO	90	–, D	6.9	16.9	0.638

## Data Availability

Data will be made available on request.
